# A Multi-Modal Convolutional Neural Network Model for Intelligent Analysis of the Influence of Music Genres on Children's Emotions

**DOI:** 10.1155/2022/4957085

**Published:** 2022-07-19

**Authors:** Qingfang Qian, Xiaofeng Chen

**Affiliations:** ^1^Conservatory of Music, Qiongtai Normal University, Haikou 571100, China; ^2^College of Teacher Education, Qiongtai Normal University, Haikou 571100, China

## Abstract

The influence of music genres on children's emotional intelligence is one of the hot topics in the field of multi-modal emotion research. How to fuse multi-modal information has an important impact on children's emotional analysis. Most of the current research is based on transformer, in which the self-attention mechanism module is improved to achieve the fusion effect of multi-modal information. However, it is difficult for these methods to effectively capture the effective information of different modalities. Therefore, for the task of the influence of music genres on children's emotions, this paper proposes a transformer-based multi-modal convolutional neural network. The first is to use the BiLSTM sub-network model to extract the video and audio features and use the BERT sub-network to extract the text features. Secondly, this paper uses the improved transformer cross-modal fusion module to effectively fuse different types of modal information. Finally, the transformer module is used to judge the information of different modalities and analyze the emotion from the multi-modal information. At the same time, a large number of experiments prove that the model based on multi-modal convolutional neural network proposed in this paper surpasses other methods in prediction accuracy and effectively improves the accuracy of sentiment classification tasks.

## 1. Introduction

With the continuous emergence of various media and short videos [1, 2] in recent years, the impact on children's emotions [3, 4] in daily life is getting bigger and bigger, such as popular music and videos published on YouTube [5, 6], TikTok [7, 8], and other platforms. Often these data contain three types of modalities, namely, video [9, 10], audio [11, 12], and text information [13]. Usually different types of music genres [14, 15] often use these three types of modalities to convey their own emotions and values, and children belong to a purely unconscious state and are easily affected by these data. Therefore, the comprehensive analysis of these multi-modal information in the fields of image processing [16, 17], natural language processing [18, 19], and audio processing [20, 21] has an important impact on the healthy growth of children.

Multi-modal information exists widely in daily life and has extremely important applications. It also has extremely important significance in the field of children's emotional analysis in music genres [22, 23]. Multi-modal learning [24, 25] involves a wide range of fields. Generally speaking, it is divided into three categories: video feature learning [26, 27], audio feature learning [28, 29], and text feature learning. Multi-modal learning aims to integrate these three categories of tasks and learn for a specific task. Therefore, there are many key points and difficulties in multi-modal learning. The first is the extraction of effective features from different types of data in multi-modality. Early research focused on hand-designed features, and subsequent work continued to use deep learning to extract effective features. The second is the fusion and joint learning of multi-modal feature information so that the neural network can effectively learn from these data.

Although the above methods have achieved certain results, the extraction and fusion learning of features of different modalities have not been fully studied, and the previous methods lead to more noise information in the model through direct feature fusion, which cannot be sufficient for learning of complementary information between modalities. With the continuous application of the attention mechanism, the researchers proposed a neural network based on transformer, which continuously strengthens the multi-modal feature information, so that the model can learn the feature information of different modalities and improve the accuracy of emotional discs.

Based on the above problems, this paper proposes a method based on multi-modal convolutional neural network to analyze the influence of music genres on children's emotions. First of all, in order to improve the network model's ability to extract different modal information, we use BiLSTM neural network and BERT neural network to extract features for video, audio, and text information, which effectively improves the feature information of multi-modalities. Secondly, based on the transformer network model, this paper adds a multi-modal information feature fusion module to perform feature fusion for different types of modal information. Finally, through the analysis of the characteristics of children's emotional analysis, certain results are achieved.

## 2. Related Work

### 2.1. Music Genre

Music is a common art form. With the continuous development of Internet technology, more types have emerged, and at the same time, it is developing in the direction of more efficient communication of digital music. The difference between different genres is difficult to distinguish by salient features, but musical works of the same genre often have similar features, and analyzing these features can bring possibilities to genre classification tasks.

Since different people have different preferences for different types of music genres, the identification technology of music genres can help music software manage and classify and push different types of music for different groups of people. Music genre identification is the cornerstone of audience management, collection, and music recommendation systems. Considering that different music has different types of styles, accompaniment instruments, singing characteristics, and so on, according to the different characteristics of the corresponding music, the characteristic indicators of the extracted music can be summarized and then divided into different music genres.

In recent years, with the continuous development of deep learning, most research works use convolutional neural network algorithms to classify music genres and achieve good prediction results. However, these algorithms have problems such as slow convergence speed and local optimum, which make it difficult to improve the accuracy and efficiency of music genre classification. To solve these problems, current deep learning-based methods mainly classify different types of music genres by rhythm, melody, harmony, sound intensity, and granularity. Different genres of music can be distinguished by the speed and strength of the music rhythm. We usually select the number of beats and the peak frequency to measure the speed and strength of the rhythm. The tune represents the pitch change, and from the perspective of the audio signal, the pitch reflects the frequency of the sound signal, that is, the frequency of the vocal cords. Usually, the expectation of the audio signal is measured to represent the pitch, the data are converted into a frequency domain signal through the Fourier base, and finally the most input data are denoised, and the average value of the music is obtained to indicate the level of the music tune. Harmony is a combination of sounds formed by the simultaneous occurrence of multiple vocal cords. It is mainly a form of superposition of the sound of musical instruments and the sound of human speech. From a data concept point of view, the domain mean analysis method describes where different instruments and vocals overlap. The sound intensity represents the acoustic properties of the event energy characteristic of the music information hall. The energy characteristics in a short time are characterized by calculating the sound intensity represented in the music information. Music, vocals, singers, and musical instruments belonging to different genres have certain differences, so it is possible to analyze music of different genres through timbre. The first is to convert the corresponding audio into a linear spectrum, then map the linear spectrum to the Mel spectrogram, and finally obtain the feature vector of the tone through Fourier transform.

### 2.2. Multi-modal Sentiment Analysis

Sentiment analysis, also known as opinion mining, is an important research direction for text mining tasks in the field of natural language processing. From the level of text analysis, sentiment analysis is divided into two levels: coarse-grained and fine-grained. Among them, attribute sentiment analysis is also called fine-grained sentiment analysis. Its main goal is to obtain the emotional attributes of the evaluation object from the text. The main task of attribute sentiment analysis is to mine the sentiment tendency corresponding to different evaluation objects in the sentence. Multimedia data consists of mainly video, audio, and text, which all have a certain degree of influence on children's emotions.

Multi-modal sentiment analysis has become one of the key researches in the field of deep learning. Its main goal is to help people to better understand the multi-modal data using feature information and pay more attention to sentiment analysis, which includes sentiment tasks such as identification and personality trait recognition. Multi-modal sentiment analysis is mainly divided into two categories, one is multi-modal data representation and the other is multi-modal data fusion.

In terms of multi-modal data representation, the methods proposed by researchers are mainly improved by novel network frameworks, data processing, and learning strategies. In terms of novel network structures, different types of network structures are usually included, and different types of sub-networks are used for different tasks. For example, two sub-network models are used in sentiment analysis tasks, one of which learns the representation features between multiple modalities, and the other network learns the features unique to each type of modalities. In terms of data processing, research work mainly focuses on using multi-modal language and vision to align multi-modal data and then obtain multi-modal data displacement methods based on these two or more modalities. In terms of learning strategies, the tasks of each sub-modality are usually designed according to different tasks, and self-supervised learning strategies are designed to improve the representation performance of the modality through multiple types of learning strategies.

In terms of multi-modal data fusion, it is usually divided into early fusion and late fusion. The early fusion method is relatively simple; it directly fuses different types of features together. Although this type of method is relatively straightforward, it can cause problems of multi-modal information imbalance, especially during model processing. The late fusion method is closer to the human method of processing multi-channel data, which can maintain the characteristics of the data while avoiding the problems caused by the feature fusion mechanism. The transformer method is a brand-new neural network model based entirely on the attention mechanism, which is divided into two parts: the encoder and the decoder. By learning a sequence of input images or audio, it can capture contextual information in the input sequence through a self-attention mechanism. BERT is a transformer network model based on bidirectional LSTM, which can effectively learn the features of masked words so that the output sequence can learn text alignment in different directions.

## 3. Multi-modal Convolutional Neural Networks

**Figure 1:**
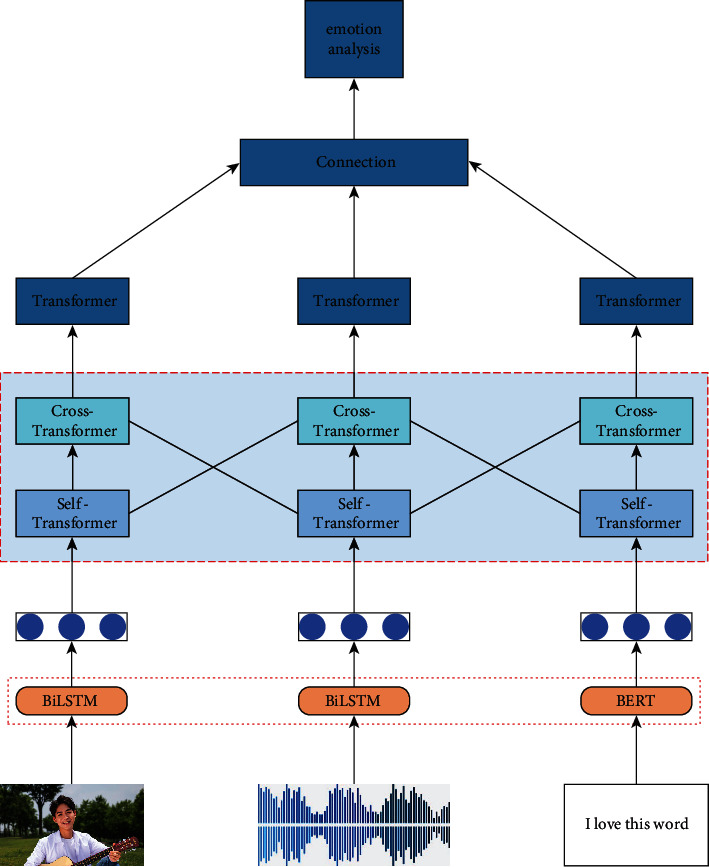
Structure diagram of multi-modal convolutional neural network.

In the multi-modal convolutional neural network, this paper uses three independent sub-networks to obtain the feature vectors represented by video, audio, and text, respectively. At the same time, this paper also uses various types of transformer modules to extract and fuse the extracted multi-modal features. In this paper, the cross-modal attention mechanism is used to continuously modulate different modal information, realize the interactive fusion of multi-modal information in time and space, and continuously improve the accuracy of the network model for emotion classification. The structure of the multi-modal convolutional neural network is shown in Figure 1. In principle, the fusion of multi-modal information can effectively improve the learning ability of the network, but the difficulty of multi-modal feature fusion lies in the alignment of features. The fusion of multi-modal data is mainly based on the input dimension of the modal data to connect.

### 3.1. Video and Audio Processing Module

**Figure 2:**
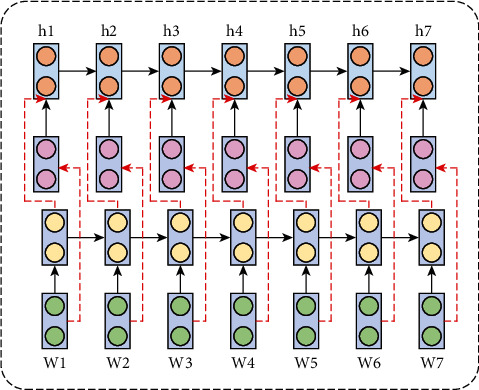
BiLSTM network structure diagram.

When performing sentiment analysis tasks, more and more researchers pay more attention to the neural network model based on deep learning to extract the characteristics of human emotion which surpasses the traditional machine learning method and has become the current mainstream research method. Sequence-based neural network models, especially LSTM technology, have been widely used in the field of sentiment analysis, and it is often used in tasks dealing with time series. The LSTM neural network solves the problems of gradient disappearance and gradient explosion caused by the increasing sequence length of the traditional RNN model by introducing a gating mechanism. As an extended model of LSTM, BiLSTM can perform bidirectional encoding representation on the input feature sequence and learn the contextual semantic information in image features. The BiLSTM network structure is shown in Figure 2. Bidirectional LSTM can encode the features of time series, that is, the later features can supplement information from the previous features. The red line indicates the direction of feature fusion by the bidirectional LSTM module.

In this paper, we use the BiLSTM network model to extract features from continuous images and audio and explore the influence of music genres on children's emotions through the follow-up attention mechanism module. In this paper, the BiLSTM neural network is used to encode the video and audio feature information, and the feature information of the context is continuously learned.

(1) $$\begin{array}{c} \overset{⟶}{h^{c}}=f_{\text{LSTM}}\;\left(\left[e_{1},e_{2},\ldots ,e_{n},\right]\right)=\left[\overset{⟶}{h_{1}^{c},}\ldots ,\overset{⟶}{h_{a_{1}}^{c},}\ldots ,\overset{⟶}{h_{1}^{c},}\right],\\ \overset{\leftarrow }{h^{c}}=f_{\text{LSTM}}\;\left(\left[e_{1},e_{2},\ldots ,e_{n},\right]\right)=\left[\overset{\leftarrow }{h_{1}^{c},}\ldots ,\overset{\leftarrow }{h_{a_{1}}^{c}}\ldots ,\overset{\leftarrow }{h_{n}^{c}}\right],\\ h^{c}=\left[\overset{⟶}{h^{c}};\overset{\leftarrow }{h^{c}}\right]=\left[h_{1}^{c},\ldots ,h_{a_{1}}^{c},\ldots ,h_{n}^{c}\right], \end{array}$$

where $\overset{⟶}{h^{c}}$ represents the forward hidden state vector sequence, $\overset{\leftarrow }{h^{c}}$ represents the backward hidden state vector sequence, *h*^*c*^ represents the hidden state vector sequence output by BiLSTM encoding, and *h*_*t*_^*c*^ belongs to the time step of the hidden state vector of *t*.

### 3.2. Text Processing Module

**Figure 3:**
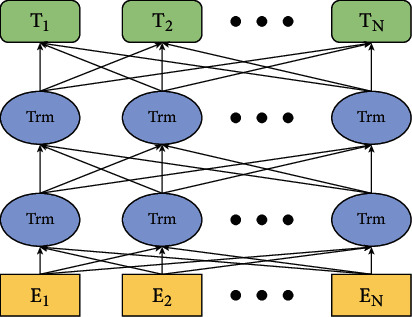
BERT network structure diagram.

We use the BERT neural network model in the text feature extraction task and feed these features together with video and audio into the attention mechanism model to classify children's emotions. The BERT network structure is shown in Figure 3. In Figure 3, Trm represents the transformer module, which mainly uses the basic LSTM module to encode text features.

BERT divides the complete word into several subsentences or words and performs random Mask operations on these separated words so that the network model can predict these masked words. This paper pays more attention to the influence of the network model on emotions and uses the BERT network model to predict the words of these emotions. When a word containing children's emotion is masked, other words belonging to this word are also randomly masked accordingly. After continuous iterative training of the BERT network model with full coverage of these words, the learning ability of the BERT network model in extracting key emotional information can be improved, and the problems of semantic ambiguity and sparse text emotional key features can be better solved. In order to enable the BERT network model to better extract the keyword features of sentiment classification operations based on the officially provided training data set, this paper establishes a data set suitable for sentiment classification for the BERT neural network. The network model is continuously optimized and improved.

The standard transformer unit in BERT mainly focuses on the modeling ability of long-distance dependencies and the global features of word vectors. In this paper, the BERT network model is used to form an encoder for feature extraction in the text emotion field. On the one hand, word vectors containing global context information can be obtained, and the loss of emotional information of global context information can be reduced. On the other hand, the global information and local analysis of the current vocabulary can be considered, and the information of children's emotions in this article can be extracted more accurately.

(2) $$\begin{array}{c} {\text{utt}}_{i,s}=\left(S_{1},S_{2},\ldots ,S_{1},\right),\\ X^{t}=\left\{x_{1}^{t},x_{2}^{t},\ldots ,x_{T^{a}}^{t},\right\},\\ \hat{X_{t}}=\text{BERT }\left(f_{\text{BERT}}\left({\text{utt}}_{i,s}\right),h\right),\\ H_{t}=\text{ReLU}\left(\hat{X}W^{f}+b^{f}\right). \end{array}$$

The original text sequence is represented by utt_*i*,*s*_; *X*^*t*^ is obtained after Tokenize processing and input into the BERT neural network model covered by global random words to obtain word vectors containing global information for training, capturing long-distance dependencies information. By activating modules and missing emotional features, a textual information *H*_*t*_ containing rich information is finally obtained. *f*_BERT_ means to tokenize the input text content. *h* is the number of hidden layers of the transformer encoder in the BERT encoder. In this paper, the model selects multiple categories of transformer modules.

### 3.3. Transmembrane State Modulation Network

In the multi-modal network structure, each type of module contributes greatly to the final sentiment analysis results. Generally speaking, audio and text have a greater impact on the final sentiment classification, while video has less impact on the classification results. Due to the large correlation between different types of module data, it is necessary to effectively fuse the feature information from different modalities to realize the feature complementarity of various types of information. In order to obtain a better fusion effect, the transformer neural network based on the attention mechanism is usually used to fuse the multi-modal information, and the attention mechanism in the transformer is improved, and the transmembrane state information is used to potentially change the different modalities and exploit the superiority of the attention mechanism model in sequence modeling. Through the modulation process, the multi-modal information from the self-network can be interactively fused in time and space to achieve the purpose of extracting complementary information.

In the neural network model of this paper, three cross-modal transformer modules are used to model video features, audio features, and text feature vectors. Each modulation module contains two transformer structures, namely, the self-transformer model and the cross-transformer module. The first is to use LSTM and BERT feature extraction network to extract video features, audio features, and text features and then use the self-transformer attention mechanism to repeatedly strengthen its own feature information cross-transformer to perform multi-modal features. Through unification, the video features, audio features, and text features are enhanced by different fusion operations between modules. Cross-modal modulation is to fuse multiple feature information, and the main problem is to align the feature information.

### 3.4. Feature Fusion Transformer Module

The self-transformer module contains two sublayers, namely, the self-attention mechanism module and the feed-forward neural module. After each core module, there is a LayerNorm layer, and there is a residual connection at the end of the module. After inputting the video features, audio features, and text features into the self-transformer sublayer, the self-attention mechanism is used to continuously strengthen its own feature representation. The feature representation of the multi-head attention mechanism is more effective and improves the effect of the attention mechanism. The calculation process of the sub-attention mechanism is as follows:

(3) $$\begin{array}{c} Q_{a}=F_{a}W_{Q},K_{a}\;=\;F_{a}W_{K},V_{a}\;=F_{a}W_{V},\\ \text{Attention}\left(Q_{a},K_{a},V_{a}\right)=\text{Softmax}\left(\frac{Q_{a}K_{a}^{T}}{\surd d_{k}}\right)V_{a},\\ W_{q}\in R^{d_{a}\times d_{k}},W_{K}\in R^{d_{a}\times d_{k}},W_{v}\in R^{d_{a}\times d_{v}}, \end{array}$$

where *W*_*q*_, *W*_*K*_, and *W*_*K*_ represent the linearly varying weight matrices of multi-modal features, respectively. For *Q*_*a*_*K*_*a*_^*T*^, the calculation result is an attention matrix, and each row represents the attention weight of the vector to itself. In order to make the surface weight too large, the magnitude of the point barricade result for the attention mechanism becomes larger, we use the Softmax function to limit the result to a small gradient range and introduce a scaling factor $\sqrt{d_{k}}$.

The main function of the cross-attention mechanism module cross-transformer lies in the application of the cross-modal attention mechanism. The specific implementation process is as follows: first, the multi-modal feature vector of the passed self-transformer module is input into the cross-transformer attention mechanism module, and the feature information is continuously enhanced. At the same time, the features from other modules, such as the video module receiving the feature information of the audio and text modules, are fused. Through the cross-modal attention mechanism, the network can fully learn different important and complementary information from three different modalities. The cross-transformer formulation of the cross-modal attention mechanism is shown as follows:

(4) $$\begin{array}{c} {\text{CM}}_{A⟶V}=\text{Softmax}\left(\frac{Q_{v}K_{a}^{T}}{\sqrt{d_{k}}}\right)V_{a}=\text{Softmax}\left(\frac{F_{v}W_{Q_{V}}W_{K_{a}}^{T}{\tilde{F}}_{a}^{T}}{\sqrt{d_{k}}}\right){\tilde{F}}_{a}W_{V_{a}},\\ W_{Q_{v}}\in R^{d_{k}\times d_{v}},\;W_{K_{a}}\in R^{d_{a}\times d_{k}},W_{V_{a}}\in R^{d_{a}\times d_{v}}, \end{array}$$

where *W*_*Q*_*v*__, *W*_*K*_*a*__, and *W*_*V*_*a*__ are the weight matrices of linear transformation. Like the subattention mechanism module, *Q*_*v*_*K*_*a*_^*T*^ calculates the attention mechanism weights from the three modal vectors, and each row represents the degree of correlation between the two modalities.

## 4. Experiments and Analysis

### 4.1. Datasets and Evaluation Metrics

In this paper, we mainly use the public dataset and the sentiment classification dataset established by ourselves to compare and test a variety of methods. The public datasets are tested on two datasets, CMU-MOSI and CMU-MOSE. These datasets contain video clips, audio clips, and corresponding text clips from YouTube; each video clip is annotated with human annotations for its sentiment classification, and the sentiment classification values range from [1–5] to represent a total of five levels.

In order to compare with other current experiments, this paper adopts a similar evaluation index. The mean absolute error (MAE) is used to measure the average absolute value of the error between the predicted value and the actual value. We use the correlation coefficient to represent the degree of correlation between the predicted value of the model and the true value. In addition to the above indicators, we also use other evaluation indicators, generally speaking, the higher the indicator, the better the performance.

### 4.2. Experimental Results and Analysis

**Figure 4:**
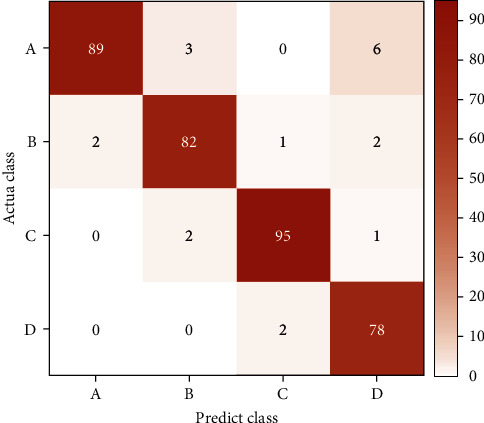
Sentiment classification confusion matrix.

In order to intuitively show the effect of the final experiment, this paper uses the confusion matrix to visualize the final results.  Figure 4 shows the confusion matrix for sentiment classification, where A, B, C, and D represent the label values of sad, a little sad, normal, and optimistic, respectively. The accuracy of the corresponding classification is shown in the data in the figure. The network model we proposed can basically achieve higher accuracy. The depth of the color in the figure represents the ratio of the number of classification accuracy. The darker the color, the better the classification effect.

**Figure 5:**
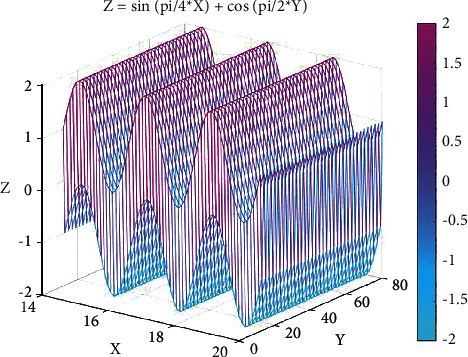
Multi-modal feature correlation degree.

Figure 5 shows the correlation degree of multi-modal features. The main variable conditions are the changes of video features, audio features, and corresponding text features. From the figure, we can see that there is a certain connection between the same video, audio, and the corresponding audio. Generally speaking, with the corresponding changes of video, audio, and text, the overall human emotion will also change.

**Figure 6:**
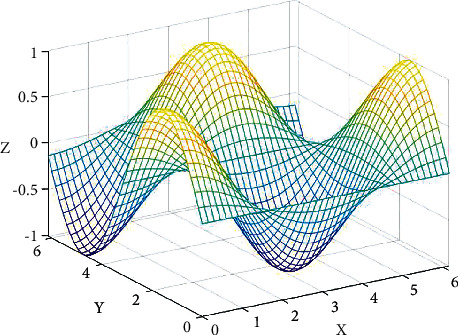
Effect of video and audio on model accuracy.

Figure 6 shows the effect of video features and text features on the accuracy of the network model. *X* represents video features, *Y* represents text features, and *Z* represents the prediction accuracy of the model. We have observed that the prediction accuracy of the model is affected to a certain extent by these two factors, and the actual prediction accuracy of the network model can be significantly improved by the correspondence between the video and the text. Within a certain range, text features and video features are aligned, which can effectively improve the actual prediction accuracy of the network model. Usually, the multi-modal data related to the same event are relatively large, and the correlation degree of different events is small.

**Figure 7:**
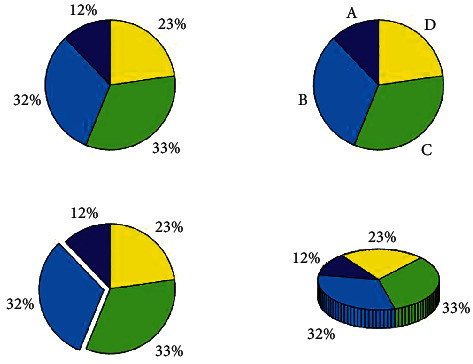
Data set collection ratio.

Figure 7 shows the proportion of data samples of children's emotions collected in this paper. From the figure, we can clearly see that the proportion of each type of sample is different, and A, B, C, and D represent four emotions, respectively. Among them, 32% of the prominent parts represent the parts that are more difficult to classify during training. Therefore, we collected some new data on the basis of the original data set and continuously expanded the data that were difficult to classify. The ability to learn and discriminate the network model can be effectively improved by continuously increasing the training data.

**Figure 8:**
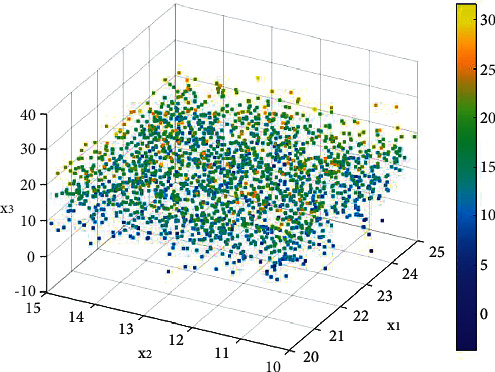
The discrete degree of sentiment in the original data.

Figure 8 shows the discrete degree of sentiment in the raw data. Among them, x1, x2, and x3 represent emotion classification, respectively. As we know, the emotion classification of human beings is difficult to understand, and there are key connections between many emotions. We analyze the labeled data of these emotions and find that there are different discrete degrees of association between these emotions.

**Figure 9:**
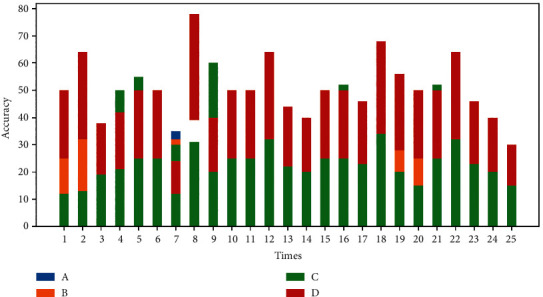
Influence of multivariate on model prediction accuracy.

In order to prove the role of the convolutional neural network-based model proposed in this paper in the emotion classification task, this paper learns and classifies different types of features, where A, B, C, and D represent video features, audio features, text features, and random noise, respectively, in Figure 9. By inputting these various types of multi-modal data, we can experimentally demonstrate the prediction accuracy of our network model.

**Figure 10:**
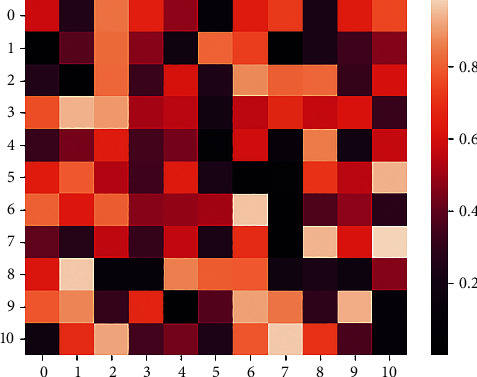
Classification of music genres.

Figure 10 shows the classification of music genres by the model. From the figure, we can see that the currently obtained music genres are classified into a total of 10 categories. The darker the color value, the worse the degree of association, and the higher the color value, the better the degree of association. We can clearly see from the figure that the classification of music genres is more difficult, much higher than the difficulty of sentiment classification.

## 5. Summary and Outlook

In order to solve the problem of multi-modal feature extraction and feature fusion, this paper proposes a multi-modal convolutional neural network, which is applied to the task of the influence of music genres on children's emotions, and improves the prediction accuracy of sentiment analysis. This paper firstly uses BiLSTM neural network to extract features for video and audio. At the same time, BERT neural network is used to extract text features. Secondly, this paper proposes an improved transformer convolutional neural network, which is added to the fusion module of multi-modal feature information and which efficiently fuses multi-modal feature information. Finally, children's emotions are judged through multi-modal feature information. Through a large number of experiments, it is proved that the method proposed in this paper surpasses the previous methods of emotion classification and has more accurate predictions on the influence of music genres on children's emotions. At the same time, this paper proposes an improved neural network model based on transformer, which can also be extended to different application scenarios, such as emotion classification, emotion prediction, and so on.

## Data Availability

The data used to support the findings of this study are available from the corresponding author upon request.
